# Perianal squamous cell carcinoma in-situ: a report of two human papilloma virus-negative cases

**DOI:** 10.1186/1757-1626-1-114

**Published:** 2008-08-20

**Authors:** James Shehan, Jeff F Wang, Susan Repertinger, Deba P Sarma

**Affiliations:** 1 Division of Dermatology, Department of Internal Medicine, Creighton University Medical Center, Omaha, NE, 68131, USA; 2 Department of Pathology, Creighton University Medical Center, Omaha, NE, 68131, USA

## Abstract

We are reporting two cases of perianal squamous cell carcinoma in-situ, negative for high-risk (HR) and low-risk (LR) human papilloma viruses. A brief review of anal and perianal squamous cell carcinoma and the role of HPV are presented.

## Introduction

Squamous cell carcinoma, the second most common form of skin cancer, most often affects sun-exposed surfaces. However, squamous cell carcinoma can involve skin surfaces not previously exposed to sunlight, such as in the anal and perianal regions. Invasive perianal squamous cell carcinoma is a locally infiltrative malignant skin tumor that exhibits destructive growth. It is a relatively uncommon tumor, which develops from the precursor lesion anal intraepithelial neoplasia (AIN). Immunosuppression is felt to be a risk factor.

## Case presentation

### Case 1

**Figure 1:**
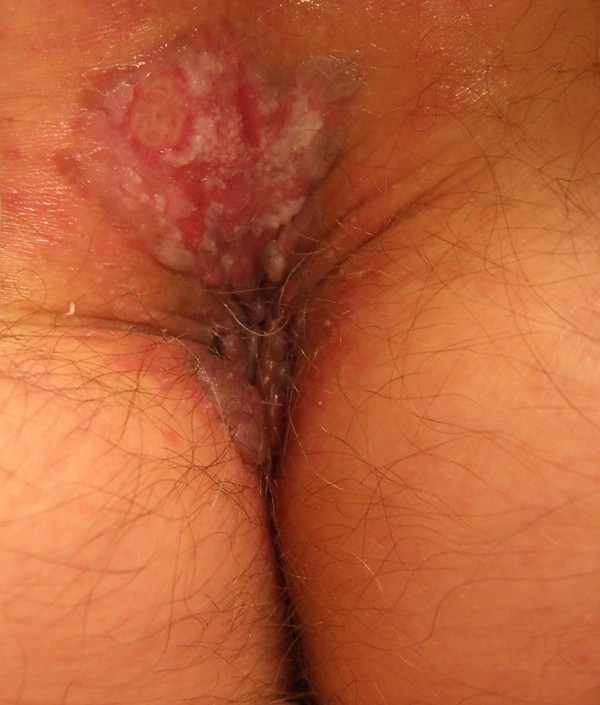
A large red-brown plaque and condyloma acuminata.

**Figure 2:**
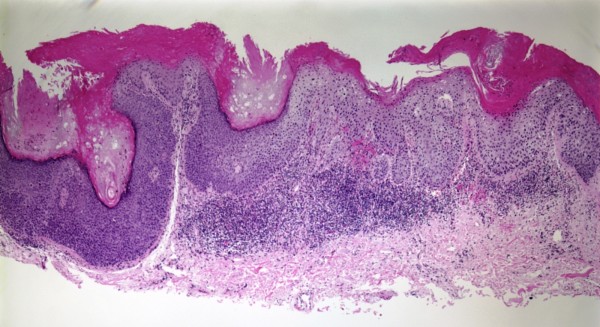
**Squamous cell carcinoma in-situ, H & E, 4×**.

**Figure 3:**
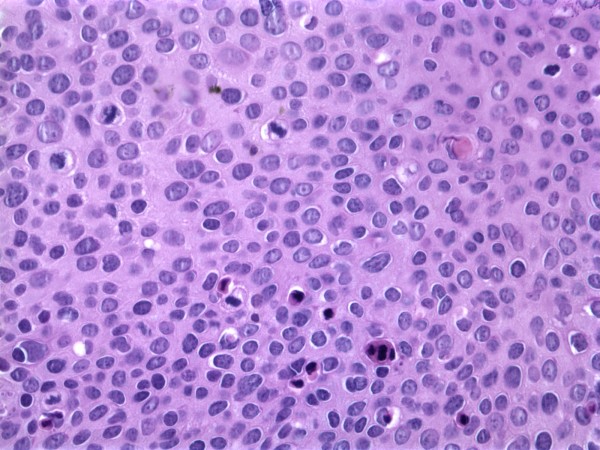
**Squamous cell carcinoma in-situ, H&E, 40×**.

A 66-year-old female was seen in the dermatology clinic for evaluation of an "irritated nevus" of the intergluteal cleft. The lesion had been present for 6 years. The patient was heterosexual with a lifetime total of 5 to 10 sexual partners. She denied a history of any sexually transmitted infections. Physical examination revealed a brown, verrucous nodule and several smaller verrucous papules in an immediate perianal distribution; these lesions were clinically consistent with condyloma acuminata. In addition, a large red-brown plaque was noted in the superior intergluteal cleft, extending to the anal verge (Figure 1). A tangential skin shave biopsy demonstrated full thickness keratinocyte dysplasia, with marked nuclear atypia and numerous mitotic figures, consistent with a diagnosis of intraepithelial squamous cell carcinoma (Figures 2, 3). Serologic testing for viral hepatitis and syphilis was negative. The patient was referred for colorectal surgery and the lesions were excised with wide margins. Severe dysplasia within the perianal area recurred 1 year later and she was treated with topical imiquimod. Further follow-up is planned.

### Case 2

**Figure 4:**
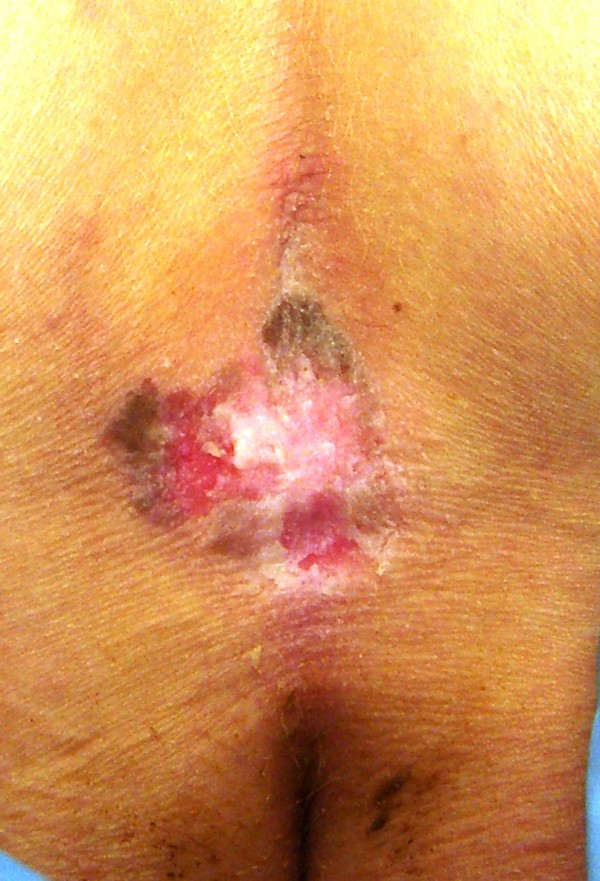
A well-demarcated red-brown plaque in superior intergluteal cleft.

**Figure 5:**
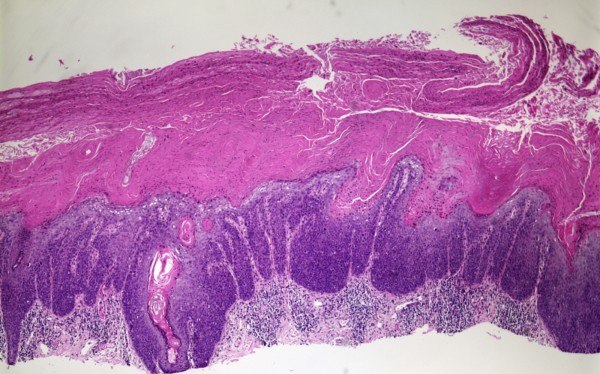
Squamous cell carcinoma in-situ, H&E, 4×.

**Figure 6:**
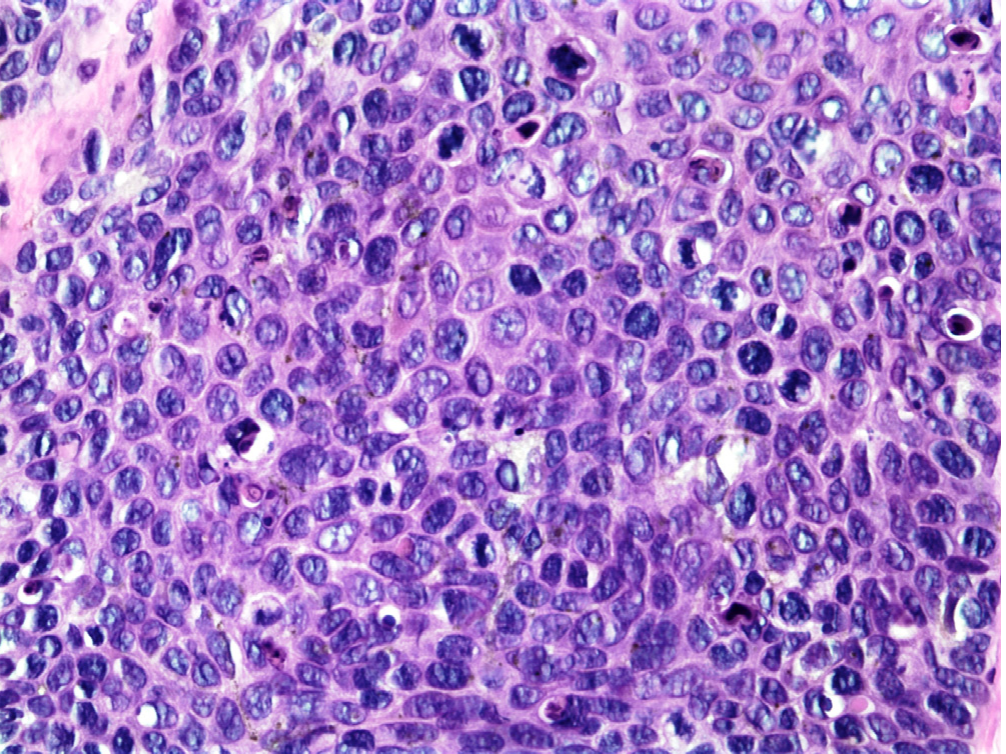
Squamous cell carcinoma in-situ, H&E, 40×.

A 60-year-old female was seen in the dermatology clinic for evaluation of a nonhealing, tender lesion in the intergluteal cleft. The lesion had been present for more than one month. The patient was heterosexual and her lifetime total number of sexual partners was unknown. Physical examination showed a well-demarcated, red-brown plaque in the superior intergluteal cleft. In contrast to the first case, this lesion did not extend any closer than 2 cm to the anal verge (Figure 4). A tangential shave biopsy was also taken in this case, and pathology revealed a non-invasive, moderately differentiated, keratinizing squamous cell carcinoma (Figure 5, 6). The patient was treated with cryotherapy and curettage destruction of the lesion. Additionally, she was referred to gastroenterology for consultation and possible proctologic examination. No additional follow-up has been available yet for this patient.

#### Special studies

Fluorescent in-situ hybridization (FISH) for high- and low-risk HPV was performed on formalin fixed, paraffin embedded tissue from each case. Both cases were negative for both categories of HPV.

## Discussion

Squamous cell carcinoma tends to develop on previously damaged skin, such as that injured by sun exposure. Immunosuppression is considered a risk factor in tumor development. Anal intraepithelial neoplasia (AIN) is a precursor lesion to invasive squamous cell carcinoma in much the same way as cervical intraepithelial neoplasia (CIN) precludes invasive carcinoma of the cervix. HPV is important in the pathogenesis of lesions in both of these sites.

More than 100 types of HPV have been reported to date. Of these, 30 infect the anogenital area. Genital HPV infection is common and usually self-limited with few, if any, symptoms. Condylomas, benign proliferative lesions, when they do occur, affect men and women and can be found on the uterine cervix; these lesions are usually associated with low-risk HPV types 6 or 11. Other HPV types that infect the anogenital region, such as high-risk types 16, 18, 31, 33, and 35, are all strongly associated with CIN. In fact, persistent infection with high-risk types of HPV is the most important risk factor for CIN and invasive carcinoma. HPV type 16 is the type most often associated with this process. Similarly, up to one-third of anal squamous cell carcinomas appear to be associated with HPV infection. Bjørge [1], in a large case-cohort study, found that patients seropositive for HPV's 16 and 18 had an increased risk of developing anal and perianal skin cancer. Perianal Bowen's disease, in particular, was associated with high risk HPV positivity [2,3].

Not all perianal squamous cell carcinoma or condylomas are associated with HPV infection, however. Frisch [4] showed fewer perianal skin cancers associated with high-risk HPV when compared to cervical and vulvar carcinomas, suggesting that in some cases, particularly in elderly patients, a causal pathway independent of HPV is present. Tachezy[5] in a series of 10 anal condylomas, found 3 to be HPV negative. Even in one of our cases, there is clear evidence of perianal condyloma acuminatum. Smoking is suggested as one risk factor that may be operative in such HPV negative cases of anal carcinoma, affecting premenopausal women more than postmenopausal women [6,7].

In our cases, we speculated that other causes such as smoking, other genital infections, or unknown factors play a role in the development of squamous cell carcinoma.

## Competing interests

The authors declare that they have no competing interests.

## Authors' contributions

JS conceived and collected the clinical data, JW drafted the manuscript, SR edited the draft, and DPS revised, formatted and submitted the manuscript.

## Consent

Written consent was obtained from the patients for publication of this report. A copy of the written consent is available for review by the Editor-in Chief of this journal.
